# Evaluation of the safety and effectiveness of crural reinforcement with bio-a^®^ or phasix-st^®^ mesh: results from a multicenter study

**DOI:** 10.1007/s10029-025-03516-3

**Published:** 2025-11-10

**Authors:** Alberto Aiolfi, Davide Bona, Sara De Bernardi, Francesca Lombardo, Michele Manara, Gianluca Bonitta, Quan Wang, Marta Cavalli, Giampiero Campanelli, Luigi Bonavina

**Affiliations:** 1 Division of General Surgery, Department of Biomedical Science for Health, I.R.C.C.S. Ospedale Galeazzi – Sant’Ambrogio, University of Milan, Via C. Belgioioso, 173, Milan, 20157 Italy; 2 Department of Biomedical Sciences for Health, Division of General and Foregut Surgery, University of Milan, IRCCS Policlinico San Donato, Milan, Italy; 3 Division of General Surgery, Department of Surgery, I.R.C.C.S. Ospedale Galeazzi- Sant’Ambrogio, University of Insubria, Milan, Italy

**Keywords:** Hiatus hernia repair, Crural reinforcement, Posterior hiatoplasty, Composite hiatoplasty, Hernia recurrence

## Abstract

**Background:**

Absorbable synthetic meshes have gained increasing acceptance for crural reinforcement during hiatus hernia (HH) repair because their safety profile and the potential of reducing recurrence rates. Bio-A^®^ (Gore Medical, Newark, DE, USA) and Phasix-ST^®^ (C.R. Bard, Inc./Davol, Inc., Warwick, RI, USA) are the most commonly used meshes. While previous single-arm studies have been published, there are no articles reporting the comparison between Phasix-ST^®^ vs. Bio-A^®^.

**Aim:**

Compare safety, efficacy, recurrence rates, and quality of life after laparoscopic HH repair and cruroplasty reinforced with either Bio-A^®^ or Phasix-ST^®^ mesh.

**Methods:**

Retrospective multicenter study (September 2011- December 2024). All patients that underwent minimally invasive HH repair with Phasix-ST^®^ or Bio-A^®^ reinforced cruroplasty and Toupet fundoplication were included.

**Results:**

Overall, 271 patients were included. Bio-A^®^ reinforcement was utilized in 46.8% of patients. The median follow-up time was 94 (IQR 21) months for Bio-A^®^ and 51 (IQR 17) months for Phasix-ST^®^ mesh. Hernia recurrence was diagnosed in 10.1% of patients with similar rates for Phasix-ST^®^ vs. Bio-A^®^ (7.8% vs. 12.6%; *p* = 0.28). The regression analysis showed that Phasix-ST^®^ (HR 0.66), ‘keyhole’ configuration (HR 0.81), hernia type III-IV (HR 1.38), and recurrent HH (HR 1.27) were not independent predictor or protective factors for recurrence. The 55-month recurrence free probability for Bio-A^®^ vs. Phasix-ST^®^ was comparable (86.2% vs. 91.8%; *p* = 0.132).

**Conclusions:**

This study shows that Bio-A^®^ and Phasix-ST^®^ are equally safe for crural reinforcement during HH repair. Due to the longer absorption rate, Phasix ST^®^ might presumably confer enhanced hiatal protection early in the course of the follow-up.

## Introduction

Minimally invasive repair is currently considered the standard of care for the management of symptomatic hiatus hernia (HH) [1, 2]. Elective surgical intervention is also recommended in asymptomatic patients presenting with large hernia defects due to the potential risk of developing serious complications, including severe postprandial discomfort, dyspnea, cardiac dysfunction, intrathoracic gastric volvulus, and/or anemia necessitating blood transfusions [3, 4]. The laparoscopic or robotic repair approach typically involves extensive esophageal dissection, adhesiolysis, hernia sac excision, tension-free cruroplasty, and fundoplication [5]. Radiological recurrence following primary repair is common, with reported incidences reaching up to 66% [6–8]. In an effort to mitigate both anatomical and clinical recurrences, various types of mesh have been utilized to reinforce the esophageal hiatus and prevent re-herniation. Although the use of non-absorbable mesh has previously demonstrated promising outcomes [9, 10], recent investigations have raised concerns regarding the potential for catastrophic complications [11, 12]. On the other hand, absorbable synthetic meshes have been associated with a reduced risk of complications and lower short- and medium-term recurrence rates when compared to simple suture repair [13]. However, the definitive role of absorbable meshes remains a subject of debate, primarily due to the paucity of long-term data [8].

Currently, two main types of fully resorbable synthetic meshes are utilized: Bio-A^®^ (Gore Medical, Newark, DE, USA) and Phasix-ST^®^ (C.R. Bard, Inc./Davol, Inc., Warwick, RI, USA). Bio-A^®^ mesh is composed of a three-dimensional polymeric web made of polyglycolic acid and trimethylene carbonate, which is gradually absorbed over a period of six months and subsequently replaced by vascularized soft tissue [14]. In contrast, Phasix-ST^®^ consists of poly-4-hydroxybutyrate (P4HB), a polymer that undergoes degradation in vivo through both hydrolysis and enzymatic processes, coated on one side with a hydrogel barrier (Sepra Technology) which can minimize adhesions to abdominal viscera. Complete resorption and full tissue incorporation of the mesh occurs within 12–18 months [15]. While previous single-arm studies have been published [14, 16–18], there are no articles comparing outcomes of Bio-A^®^ vs. Phasix-ST^®^ for crural reinforcement during HH repair.

The present study aims to compare safety, efficacy, recurrence rates, and quality of life after laparoscopic HH repair and cruroplasty reinforced with either Bio-A^®^ or Phasix-ST^®^ mesh.

## Materials and methods

### Study design

Observational multi-center retrospective cohort study carried out at two specialized esophageal cancer centers in Italy (IRCCS Policlinico San Donato and IRCCS Ospedale Galeazzi-Sant’Ambrogio). The study was approved by the locals Institutional Review Board and performed in accordance with the Declaration of Helsinki. Written informed consent was obtained from all patients. All patients with primary or recurrent symptomatic HH undergoing elective laparoscopic repair were entered into a prospectively maintained database. The dataset was supported by a cloud-based collaborative platform, securely stored on a cloud server, and safeguarded with unique password protection. We retrospectively queried the database to identify all adult patients (≥ 18 years old) who underwent elective laparoscopic repair with Bio-A^®^ or Phasix-ST^®^ mesh as an adjunct to Toupet fundoplication from September 2011 to December 2024. The implantation of Bio-A^®^ mesh began in September 2011 and was gradually discontinued in routine clinical practice following the introduction of the Phasix-ST^®^ mesh in January 2017.

Patients with missing operative reports or follow-up data, who underwent fundoplication other than Toupet, who underwent emergency repair, or who underwent HH repair without mesh reinforcement were excluded from study. All patients underwent a standard preoperative assessment including medical history, disease-specific and generic quality of life evaluation, chest X-ray, barium swallow study, and upper gastrointestinal endoscopy with biopsies. In selected patients, high-resolution manometry and CT scan of chest and upper abdomen were also performed.

## Surgical technique

The patient was positioned in the reverse Trendelenburg orientation, with the primary surgeon standing between the patient’s legs. Five trocars were utilized for the procedure. Following mediastinal dissection and excision of the hernia sac, pneumoperitoneum was established at 8 mmHg. Posterior cruroplasty was then performed using 3 to 4 interrupted, 2 − 0/0 Prolene^®^ sutures), calibrated visually so that the hiatus was in loose contact with the esophagus. One or 2 left-lateral sutures including the central tendon of the diaphragm were placed if the hiatus could not be adequately approximated with just the posterior sutures. A 7 × 10 cm Bio-A^®^ mesh, pre-shaped in a U-configuration, was placed to cover the approximated hiatus and secured with 2 or 3 nonabsorbable sutures. Since January 2017, a 7 × 10 cm resorbable Phasix-ST^®^ mesh was shaped into a U- or keyhole circumferential configuration and fixed with 2–3 absorbable sutures over the approximated hiatus, with the hydrogel ST barrier facing the abdominal side. Since January 2022, we moved to a ‘keyhole’ circumferential configuration with the intent to reinforce also the central tendon and the left-lateral part of the hiatus [19, 20].

Upon division of the upper short gastric vessels, a 270° Toupet fundoplication was fashioned in all patients. To prevent postoperative nausea and vomiting, intravenous Ondansetron (4 mg) and Dexamethasone (8 mg) was administered intraoperatively. The nasogastric tube was routinely removed at the end of the procedure. On postoperative day one, a chest radiograph and a gastrographin swallow study were routinely performed. Patients were then advanced to a soft diet and subsequently discharged.

## Follow-up

Outpatient follow-up visits were scheduled at 1, 6, and 12 months postoperatively, and annually thereafter. Routine barium swallow study was conducted at 6 months after surgery and upper endoscopy at 12 months. After 12 months, if the patient remained asymptomatic, no additional investigations were performed [21]. Upper endoscopy and/or barium swallow studies were performed whenever patients reported recurrent symptoms or for investigational purposes. If a patient missed the annual follow-up visit, she/he was contacted by telephone or email to inquire about any recurrent symptoms and quality of life. Patients whose telephone was disconnected, who did not respond after repeat call attempts, or failed to reply to repeat email attempts were classified as lost to follow-up. The follow-up was updated in June and July 2025.

## Outcomes

The primary outcome was postoperative HH recurrence. Secondary outcomes were short-term postoperative complications, postoperative proton pump inhibitor (PPI) use, and quality of life assessment with disease-specific and generic questionnaire. Recurrence was defined as the presence of recurrent GERD symptoms (heartburn, regurgitation, dysphagia, chest pain) and more than 2 cm of gastric tissue herniating above the diaphragmatic impression on follow-up upper endoscopy and/or barium swallow study [22]. Perioperative complications were classified according to the modified Clavien–Dindo (CD) classification [23]. Disease-specific Gastro-Esophageal Reflux Disease Health-Related Quality of Life (GERD-HRQL) and generic Short Form-36 (SF-36) questionnaires were administered at baseline and during follow-up visits to assess patient quality of life.

The GERD-HRQL questionnaire is a disease-specific instrument comprising 10 items that assess symptoms related to heartburn, dysphagia, and gas bloat [24]. Each symptom is rated on a scale from 0 to 5, with the total score representing the sum of individual item scores. Consequently, an asymptomatic patient would have a total score of 0, whereas the maximum possible score of 50 indicates the most severe symptomatology. A score below 10 is generally considered within the normal range. Additionally, the questionnaire includes a separate item not incorporated into the total score calculation that evaluates overall current patient satisfaction.

The SF-36 Health Survey is a comprehensive, multidimensional instrument consisting of 36 items designed to evaluate health-related quality of life across eight distinct domains: physical functioning (10 items), role limitations due to physical health (4 items), bodily pain (2 items), general health perceptions (5 items), vitality (4 items), social functioning (2 items), role limitations resulting from emotional problems (3 items), and mental health (5 items) [25]. Each domain score is standardized on a scale from 0 to 100, with higher scores indicating better health status. Composite scores known as the Physical Component Summary (PCS) and Mental Component Summary (MCS) are also calculated, ranging from 0 (lowest well-being) to 100 (highest well-being). The summary scores are derived from weighted combinations of the eight domain scores, with factor weights obtained via both orthogonal and oblique factor rotation methods. Orthogonal rotation yields factor weights that minimize the correlation between PCS and MCS, whereas oblique rotation permits correlation between these composite measures.

### Statistical analysis

Categorical variables were presented as absolute and percentage frequencies, while continuous variables were expressed as median values along with interquartile ranges (IQR). The Mann-Whitney U-test or Chi-Square test were performed as appropriate to compare Bio-A^®^ and Phasix-ST^®^. Multivariable Cox regression analysis including its diagnostics was conducted to identify independent predictors of HH recurrence. The linear predictors include age, type of hernia, recurrent HH, type of mesh, and mesh shape. Kaplan-Meier survival analysis was performed to estimate the time to recurrence along with 95% confidence intervals (95% CI). Difference in Kaplan-Meier curves were assessed with the Log Rank test. Statistical significance was defined as *p* < 0.05 (α). The statistical analysis was conducted using R software version 3.2.2 from the R Foundation in Vienna, Austria 20 [26].

## Results

During the study period 2011–2024, 952 patients with HH were operated in our centers. Overall, 271 patients who underwent hiatoplasty reinforced with Bio-A^®^ or Phasix-ST^®^ and Toupet fundoplication were considered for final analysis. Bio-A^®^ and Phasix-ST^®^ mesh were used in 127 (46.8%) and 144 patients (53.2%), respectively. The demographic characteristics and preoperative clinical data of the study population are summarized in Table 1. The prevalence of patients undergoing repair for recurrent HH was significantly greater in the Phasix-ST^®^ group (*p* = 0.004).

**Table 1:** Baseline, demographics, comorbidities, symptoms and preoperative endoscopic findings. ASA score American society of anesthesiologists score; BMI body mass index; CAD coronary artery disease; COPD chronic obstructive pulmonary disease; GERD-HRQL GERD-Health related quality of Life. Data are presented as median (interquartile range – IQR) or numbers (percentage)

	Bio-A^®^ (*n* = 127)	Phasix-ST^®^ (*n* = 144)	*p* value
Age, years, median (IQR)	67.1 (12.1)	67.8 (15.3)	0.545
Gender, female, *n* (%)	103 (81.1)	119 (82.6)	0.865
ASA score, median (IQR)	2 (1)	3 (1)	0.999
BMI, kg/m2, median (IQR)	26.9 (4.4)	27.3 (5.1)	0.642
Comorbidities			
Hypertension, *n* (%)	72 (56.7)	79 (54.8)	0.856
Diabetes, *n* (%)	22 (17.3)	15 (10.4)	0.140
Smoke, *n* (%)	29 (22.8)	33 (22.9)	0.999
CAD, *n* (%)	14 (11)	18 (12.5)	0.851
COPD, *n* (%)	12 (9.4)	15 (10.4)	0.950
Kyphoscoliosis, *n* (%)	29 (22.8)	28 (19.4)	0.593
Symptoms			
Heartburn, *n* (%)	80 (63)	93 (64.6)	0.884
Regurgitation, *n* (%)	71 (55.9)	82 (56.9)	0.960
Chest pain, *n* (%)	52 (40.9)	61 (42.7)	0.910
Dyspnea, *n* (%)	38 (29.9)	47 (32.6)	0.726
Dysphagia, *n* (%)	39 (30.7)	50 (34.8)	0.567
Anemia/previous blood transfusion, *n* (%)	22 (17.3)	27 (18.7)	0.883
Nausea/vomiting, *n* (%)	15 (11.8)	18 (12.5)	0.999
Symptom duration, years, *n* (%)	5.2 (3.1)	5.6 (4.7)	0.167
GERD-HRQL, median (IQR)	17.2 (7.1)	16.8 (6.3)	0.433
Hernia type			
Type I, *n* (%)	12 (9.4)	9 (6.2)	0.450
Type II, *n* (%)	3 (2.3)	1 (0.7)	0.527
Type III, *n* (%)	97 (76.5)	113 (78.4)	0.790
Type IV, *n* (%)	15 (11.8)	21 (14.7)	0.623
Recurrent hiatal hernia	28 (22.1)	56 (38.8)	0.004
Endoscopic findings			
Esophagitis, *n* (%)	21 (16.5)	26 (18)	0.865
Barrett esophagus, *n* (%)	11 (8.7)	11 (7.6)	0.932
Hernia size, cm, median (IQR)	7.3 (3.1)	6.9 (4.8)	0.352

All the operations were completed laparoscopically. The median operative time was comparable in the two groups. Posterior cruroplasty was performed using a median of 4 stitches; additional stitches for left crus/central tendon approximation were deemed necessary in 61 patients (22.5%). The method for mesh fixation was similar among the two groups, while a ‘keyhole’ configuration was significantly more prevalent in the Phasix-ST^®^ group (15.9% vs. 0%; *p* = 0.001). No intraoperative complications were reported. Median blood loss and length of hospital stay were comparable between groups. The overall postoperative complication rate was 11.4% (*n* = 31), with no statistically significant difference observed between groups. One patient in the Phasix-ST^®^ group died postoperatively due to cardiac arrest (CD grade V). Importantly, the 90-day hospital readmission rates were similar (Table 2).

**Table 2:** Postoperative outcomes comparison

	Bio-A^®^ (*n* = 127)	Phasix-ST^®^ (*n* = 144)	*p* value
Estimated blood loss, ml, median (IQR	105 (65)	90 (80)	0.339
OT, minutes, median (IQR)	158 (67)	139 (85)	0.084
Posterior cruroplasty, median (IQR)	4 (2)	4 (2)	0.999
Left-anterior cruroplasty, *n* (%)	26 (20.5)	35 (24.3)	0.543
Left-anterior cruroplasty, median (IQR)	1 (2)	1 (2)	0.999
U-shaped mesh, *n* (%)	127 (100)	121 (84.1)	< 0.001
Keyhole shaped mesh, *n* (%)	0 (0)	23 (15.9)	< 0.001
ICU stay, days, median (IQR)	1 (1)	0 (1)	0.842
HLOS, days, median (IQR)	3 (2)	3 (1)	0.887
Overall morbidity CD *≥* II, *n *(%)	16 (12.6)	15 (10.4)	0.710
CD *≥* IIIB, *n* (%)	2 (1.5)	3 (2.1)	0.898
90-day hospital readmission, *n* (%)	3 (2.3)	3 (2.1)	0.997
Endoscopic dilation, *n *(%)	3 (2.5) ^*^	2 (1.4) ^♦^	0.798
Recurrence, *n* (%)	15 (12.6) ^*^	11 (7.8) ^♦^	0.289
Early (< 12-month) recurrence, *n* (%)	2 (13.3)	1 (9.1)	0.912
Late (> 12 month) recurrence, *n* (%)	13 (86.7)	10 (90.9)	0.452
PPI off, *n* (%)	100 (84.2) ^*^	120 (85.7) ^♦^	0.839
GERD-HRQL, median (IQR)	4.2 (2.3) ^*^	3.1 (2.9) ^♦^	0.235
Redo surgery, *n* (%)	2 (1.7) ^*^	2 (1.4) ^♦^	0.887

OT operative time; ICU intensive care unit; HLOS hospital length of stay; CD Clavien-Dindo. data are presented as median (interquartile range – IQR) or numbers (percentage). * data based on 119 patients that completed the last follow-up visit/interview. ^♦^ data based on 140 patients that completed the last follow-up visit/interview

**Fig. 1:**
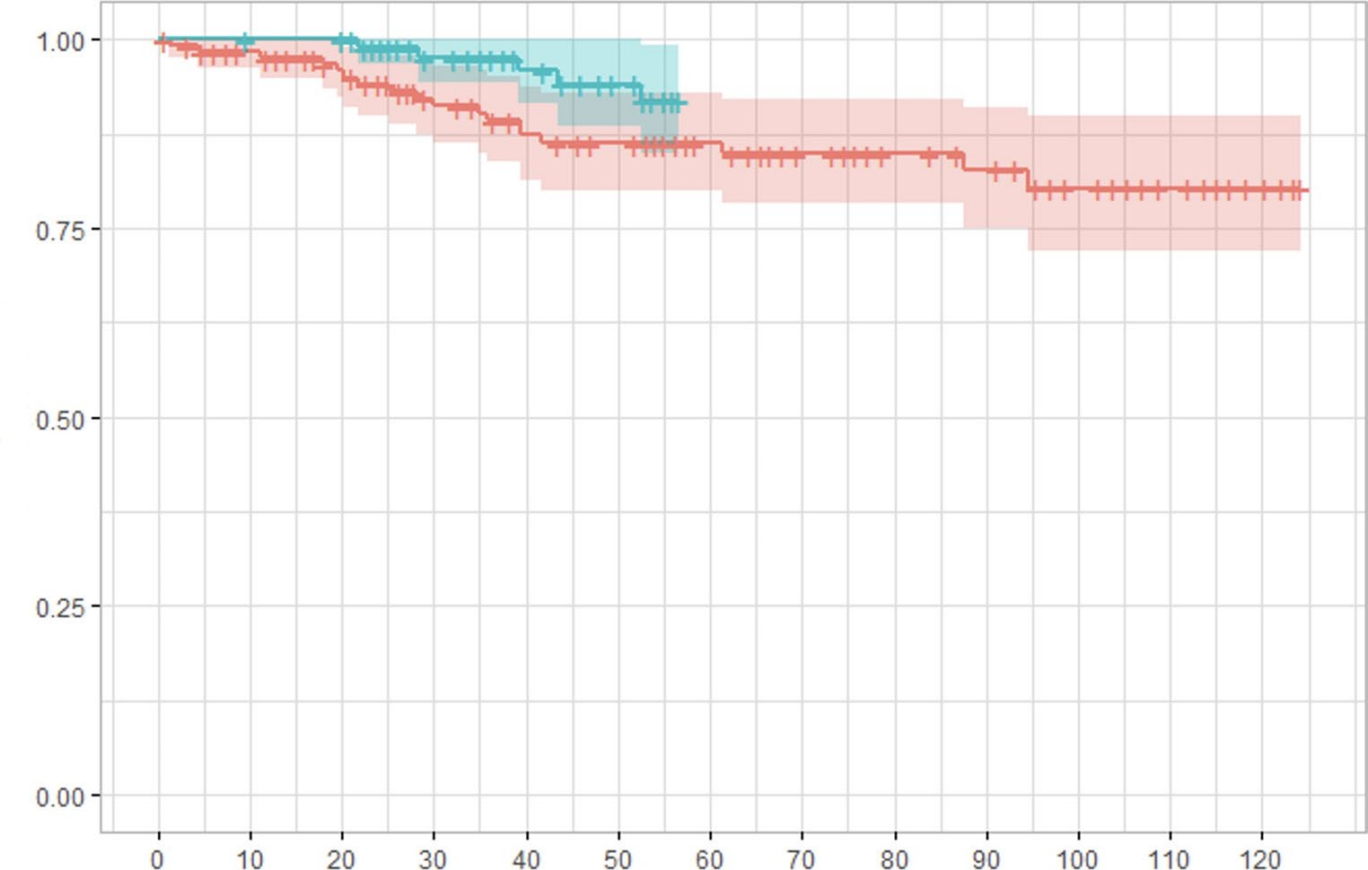
Kaplan-Meier survival curve for hernia recurrence comparing Phasix-ST^®^ (green line) vs. Bio-A^®^ (red line) mesh. The continuous line represents the median while the green and red shadow represent confidence intervals. The X axis represent postoperative follow-up time (months). The Y axis represent the recurrence probability

All included patients completed a minimum follow-up of 6 months. The median follow-up time was 94 (IQR 21) months for Bio-A^®^ and 51 (IQR 17) months for Phasix-ST^®^ mesh. Four patients died during follow-up due to unrelated causes, and 8 patients were lost to follow-up. As a result, 259 patients completed their final outpatient visit or interview. Hernia recurrence was diagnosed in 26 patients (10.1%) with lower recurrence rates in the Phasix-ST^®^ group (7.8% vs. 12.6%; *p* = 0.289). The regression analysis adjusted for relevant clinical factors showed that Phasix-ST^®^ mesh (HR 0.66; 95% CI 0.26–1.22), ‘keyhole’ mesh configuration (HR 0.81; 95% CI 0.59–1.74), type III-IV HH (HR 1.38), and recurrent HH (HR 1.27) were not independent predictors or protective factors of HH recurrence (Table 3). There was no evidence against violation of proportional Hazard assumption (Schoenfield residual based statistics *p* = 0.751). Postoperative recurrences were observed between 3 and 41 months (median 14 months) in the Bio-A^®^ group and between 9 and 36 months (median 23 months) in the Phasix-ST^®^ group. The 55-month recurrence free probability for Bio-A^®^ (86.2%; 95% CI 80–93%) vs. Phasix-ST^®^ (91.8%; 95% CI 85–99.2.2%) was comparable (*p* = 0.132) (Fig. 1). Most recurrences (88.5%) were documented 12-month after surgery. Overall, 4 out of 26 (15.3%) patients with HH recurrence required surgical revision because of uncontrolled symptoms, with no difference among groups.

**Table 3:** Cox regression model for HH recurrence. 95% CI confidence intervals

		Hazard Ratio	95% CI	*P* value
Age (years)		1.03	.56-39	0.689
Hernia type				
	I-II	Ref		
	III-IV	1.38	0.95–2.03	0.743
Recurrent HH		1.27	0.76–1.64	0.641
Mesh				
	Bio-A^®^	Ref		
	Phasix-ST^®^	0.66	0.26–1.22	0.241
Shape				
	U-shape	Ref		
	Keyhole	0.81	0.59–1.74	0.852

No mesh-related complications were detected during follow-up. Postoperative dysphagia requiring one session of endoscopic dilation occurred in five patients (1.8%) with no differences between Bio-A^®^ and. Phasix-ST^®^ (2.3% vs. 1.4%; *p* = 0.798). At the last follow-up, most patients were off PPI with no difference between Bio-A^®^ vs. Phasix-ST^®^ (84.2% vs. 85.7%; *p* = 0.839). Compared to baseline, the GERD-HRQL score (*p* < 0.001) and all SF-36 items (*p* < 0.001) significantly improved in both groups. No significant differences among groups were found in terms of postoperative GERD-HRQL score (*p* = 0.881), but patients in the Phasix-ST^®^ group showed improved role physical function (82.1 vs. 74.7; *p* = 0.021), bodily pain score (81.2 vs. 75.6; *p* = 0.03), and social function (83.4 vs. 69.2; *p* = 0.017). Finally, both the physical (78.2 vs. 71.4; *p* = 0.01) and mental (78.8 vs. 72.3; *p* = 0.01) component summary scores were significantly higher in the Phasix-ST^®^ group (Table 4).

**Table 4:** Baseline and postoperative results of the Short-Form 36 (SF-36) questionnaire. Values are expressed as median (interquartile range). * data based on 119 patients that completed the last follow-up visit/interview. ^♦^ data based on 140 patients that completed the last follow-up visit/interview

	Pre		*p* value	Post		*p* value
	Bio-A^®^	Phasix-ST^®^		Bio-A^®^*	Phasix-ST^®^^♦^	
Physical function (PF)	46.1 (18.9)	44.9 (20.2)	0.09	68.7 (13.2)	70.4 (10.2)	0.086
Role physical (RP)	48.1 (21.7)	50.4 (22.4)	0.26	74.7 (20.1)	82.1 (22.6)	0.021
Bodily pain (BP)	43.9 (14.1)	40.4 (13.7)	0.19	75.6 (19.3)	81.2 (17.9)	0.025
General health (GH)	44.5 (17.1)	43.2 (15.9)	0.29	60.9 (14.3)	64.2 (15.9)	0.12
Vitality (VT)	47.3 (19.9)	44.1 (20.4)	0.07	67.4 (15.2)	68.1 (13.6)	0.43
Social function (SF)	45.1 (18.7)	47.4 (20.2)	0.21	69.2 (14.9)	83.4 (16.6)	0.017
Role emotional (RE)	49.1 (21)	51.1 (18.5)	0.34	77.1 (18.2)	79.1 (20.3)	0.26
Mental health (MH)	59.4 (18.2)	62.2 (19.7)	0.18	83.1 (15.1)	85.2 (19.1)	0.31
Component summary (orthogonal rotation weight)						
Physical (PCS)	33.4 (21.2)	36.6 (22.5)	0.13	69.7 (21.4)	75.4 (22.6)	0.024
Mental (MCS)	52.7 (19.2)	55.8 (25.1)	0.25	71.6 (19.7)	76.9 (23.7)	0.037
Component summary (oblique rotation weight)						
Physical (PCS)	46.9 (20.3)	48.4 (21.6)	0.58	71.4 (23.4)	78.2 (22.2)	0.012
Mental (MCS)	55.3 (19.8)	56.7 (17.8)	0.44	72.3 (20.3)	78.3 (18.2)	0.019

## Discussion

Our study shows that Bio-A^®^ and Phasix-ST^®^ mesh are equally safe for crural reinforcement during HH repair, and use of either mesh appeared to minimize recurrence rates and improve quality of life over an extended follow-up period.

The use of mesh for reinforcement in HH repairs remains a contentious topic. Two recent meta-analyses of randomized controlled trials (RCTs) reported no clear advantage of mesh augmentation compared to standard crural suturing [27, 28]. However, the heterogeneity in study parameters—inclusion criteria, hernia size, surgical indications, definitions of recurrence, surgeon experience, mesh properties, methods of crural fixation, and types of fundoplication—complicates the interpretation of these findings. In contemporary clinical practice, up to 80% of surgeons consider performing mesh-reinforced cruroplasty for paraesophageal hernia repair [29]. Additionally, recent epidemiological data from Europe and North America indicate that a mesh is used in approximately 35% of the laparoscopic procedures for PEH [30–32]. Currently, no guidelines exist and the decision behind hiatal reinforcement is largely influenced by individual surgeon attitude and the intraoperative ‘feeling’ of crural weakness [33]. This subjectivity introduces further interobserver variability and heterogeneity, which substantially limit the conclusiveness of prior RCTs [34]. Since the initial application of a polyester mesh as a bridging hiatal repair in 1993 [35], additional studies have documented the use of polypropylene and polytetrafluoroethylene (PTFE) mesh as an onlay reinforcement [36]. However, the reported risk of serious complications—such as erosion into the stomach or esophagus—has limited the widespread adoption of these materials [12]. Consequently, absorbable biological and biosynthetic meshes were introduced to retain the theoretical advantage of reducing recurrence rates while avoiding the morbidity linked to non-absorbable meshes [8, 37].

Currently, Bio-A^®^ and Phasix-ST^®^ remain the most commonly used fully resorbable synthetic mesh for crural reinforcement. The clinical outcomes associated with these materials have primarily been documented through single-arm studies, whereas direct comparative data are lacking. Abdelmoaty et al. retrospectively evaluated the use of Phasix-ST^®^ in 90 consecutive patients with PEH, observing no mesh-related adverse events and 8% recurrence rate at a median follow-up of one year [16]. Similarly, a retrospective study involving 68 consecutive PEH patients who underwent crural reinforcement with Phasix-ST^®^ reported a recurrence rate of 8.8% over a median follow-up period of 27 months, with no associated adverse events [38]. In another single-center retrospective analysis, Armijo and colleagues assessed 83 patients with HH treated with Bio-A^®^, documenting a recurrence rate of up to 17% at 27 months postoperatively, without any mesh-related complications [7].

The present study is the first to report comparative outcome data between Bio-A^®^ and Phasix-ST^®^. The overall recurrence rate was comparable between groups on univariate analysis (7.8% vs. 12.6%; *p* = 0.289). However, although the logistic regression analysis yielded a hazard ratio of 0.66 for Phasix-ST^®^, suggesting a potential protective effect, the 95% confidence interval included the null value, indicating a lack of statistical significance. The observed lack of statistical significance may be explained by the relatively low number of recurrences within the cohort, which limits the power to detect meaningful differences between groups. The distinct polymer composition of Phasix-ST^®^, with a trend toward longer fully resorption time (12–18 months) may account for the better early outcomes. It is indeed important to distinguish between early and late HH recurrences [17]. Notably, most recurrences were diagnosed after 12-month from the surgical procedure. Previous retrospective studies indicate that most earlier recurrences are typically due to disruption of the crural repair and are posterior or circumferential [19, 20]. These cases are best understood as technical failures. Conversely, recurrences that develop after longer follow-up periods tend to arise at the anterior or left-lateral aspect of the hiatus and may rather indicate progressive disease and ongoing physiological stress on the repair leading to gradual stretching and widening of this most vulnerable anatomical area [19]. For these reasons, we introduced two changes in our surgical technique after 2020. Firstly, we paid more attention to the symmetry of suture repair of the hiatus and to the theoretical distribution of the vectors of radial force which may be underestimated; therefore, in addition to the posterior sutures, we protected the anterior-left-lateral sector of the hiatus with 1–2 additional stitches. Secondly, we changed the shape of the mesh to a ‘keyhole’ circular configuration with the intent to further protect the anterior and left-lateral portion of the hiatus. Although logistic regression analysis did not show a statistically significant effect of mesh configuration on recurrence risk, the point estimate was < 1, which suggests a potential clinical benefit (HR 0.81). This may suggest that circular mesh placement has the potential to reduce recurrence rates compared to the U-shaped, posterior mesh configuration [17, 39]. Whether the combination of left-anterior sutures and mesh is necessary to provide optimal results remains to be determined.

Symptomatic control in patients with HH recurrence was generally achieved with PPI therapy in 22 patients, and only 4 individuals (15.3%) required surgical revision with no significant difference observed between Phasix-ST^®^ and Bio-A^®^. These results are consistent with prior research, including studies by Lidor et al. and Wang and colleagues, which also documented low reoperation rates for HH recurrence [40]– [41]. Additionally, postoperative endoscopic dilation for dysphagia rates did not differ significantly between the Bio-A^®^ and Phasix-ST^®^ cohorts (2.5% vs. 1.4%). Notably, none of the patients was diagnosed with esophageal stricture. Quality of life in patients with HH may be severely disrupted to the point of affecting everyday activities, social functioning, and mental health [42–44]. Compared to baseline, we found a significant improvement (>50% from baseline) in GERD-HRQL and SF-36 scores in both groups. These data are in line with a recent systematic review [18]. Notably, Phasix-ST^®^ patients showed significantly higher improvement in both physical and mental component summaries with improvement of physical limitations (role-physical), suffered pain (bodily pain), and ability to participate in social activities (social functioning). Although we recognize that subjective, cultural, and social factors may affect these results, particularly when analyzing SF-36 results [45], we speculate that these findings might be associated with the trend indicating a reduced incidence of recurrence.

It is necessary to emphasize that hiatal mesh reinforcement does not obviate the need for a meticulous surgical technique [46, 47]. Adequate esophageal mediastinal dissection is critical to achieve a minimum of 3 cm of intra-abdominal esophagus without tension [48]. Moreover, a careful assessment of hiatal geometry is particularly important in older patients with fragile crura [49–51]. Appropriate crural approximation and a composite repair might be required especially in individuals with oval ort round-shaped hiatus [52–57]. Hence, while mesh reinforcement of the crura may further minimize recurrence rates, it does not replace the necessity for a precise suture hiatoplasty. Additionally, division of the proximal short-gastric vessels is necessary in our opinion to secure a tension-free, symmetric valve and prevent twisting of the distal esophagus [33, 48]. It seems obvious that the experience of the operating surgeon plays a major role in determining the long-term outcomes of any type of hiatal repair [58, 59]. In the future, developments in artificial intelligence and of autologous biological therapies such as Platelet Rich Plasma (PRP) may contribute to further reduce the HH recurrence rates [60–65].

Up to our knowledge this is the first report describing post-operative outcomes comparing Bio-A^®^ and Phasix-ST^®^ biosynthetic absorbable mesh. A notable strength of this study is that all procedures were conducted by experienced surgeons specialized in upper gastrointestinal surgery, with patients consistently receiving special attention to assessment of hiatal geometry and the same type of fundoplication. Additionally, use of a standardized definition for hiatal hernia recurrence contributes to methodological rigor. Nevertheless, all limitations related to the study design should be considered for interpreting our findings, especially selection and temporal bias. Additionally, inter-operator variability ought to be acknowledged as a potential confounding factor, even when all procedures are carried out by expert foregut surgeons within referral centers. The median follow-up period was longer in the Bio-A^®^ group, suggesting that additional HH recurrences may emerge in the Phasix-ST^®^ group over time. Interestingly, in the Phasix-ST^®^ group, timing of recurrence spanned between 9 and 36 months. These observations might suggest that recurrence events may predominantly cluster within these intervals, with limited likelihood of further episodes during extended follow-up. It is unclear whether this represents a coincidence or a clinically significant observation. Although patients lost to follow-up comprised less than 20% of the total study population, the potential for attrition bias cannot be excluded. It is plausible to hypothesize that these patients may have experienced a greater incidence of adverse effects or higher recurrence rates, which could lead to an overall underestimation of our outcomes. Finally, the limited recurrence events may impact the robustness of regression analyses.

## Conclusions

Bio-A^®^ and Phasix-ST^®^ are equally safe for use in crural reinforcement during HH repair. Due to the longer absorption rate, Phasix ST^®^ may confer greater hiatal protection early in the course of the follow-up. However, caution is warranted in interpreting our findings and studies with longer follow-up are mandatory to clarify the controversies surrounding the use of current absorbable mesh in hiatal surgery.

## Data Availability

The authors confirm that the data supporting the findings of this study are available upon reasonable request.

## Declarations

### Declarations of generative AI and AI-assisted technologies in the writing process

During the preparation of this work the authors did not use any tool or service to modify/adapt/revise its content.
